# Vestibular dysfunction in lateral semicircular canal dysplasia

**DOI:** 10.3389/fneur.2024.1341812

**Published:** 2024-01-17

**Authors:** Ji Min Yun, Sung Huhn Kim, Seong Hoon Bae

**Affiliations:** ^1^Department of Otorhinolaryngology, Severance Hospital, Yonsei University College of Medicine, Seoul, Republic of Korea; ^2^Department of Otorhinolaryngology, Gangnam Severance Hospital, Yonsei University College of Medicine, Seoul, Republic of Korea

**Keywords:** semicircular canals, vestibular function tests, head impulse test, caloric tests lateral, inner ear malformation

## Abstract

**Introduction:**

Lateral semicircular canal (LSCC) dysplasia is the most common inner ear malformation. The severity of dysplasia can appear in various spectrums, from a short and broad LSCC with normal or small-sized central bony island (CBI) to a single fluid-filled cavity confluent with the vestibule without CBI. However, reports on the association between LSCC dysplasia and the loss of vestibular function are still lacking. In this study, the results of vestibular function tests [caloric test and video-head impulse test (vHIT)] in patients with LSCC dysplasia were analyzed and compared between groups with and without CBI.

**Methods:**

This study retrospectively enrolled 17 patients (23 ears) who had LSCC dysplasia following computed tomography or magnetic resonance imaging and underwent vestibular function tests.

**Results:**

LSCC dysplasia was observed unilaterally in 11 patients and bilaterally in six patients. Nine of 23 ears had CBIs, and 14 ears had no CBI. Three of 17 patients experienced dizziness. Abnormal caloric tests were detected in 11 of the 16 patients who underwent the caloric tests (69%); in contrast, 11 of 12 patients who underwent the vHIT (92%) had normal LSCC vestibulo-ocular reflex (VOR) gain on vHIT. A significant correlation was found between the maximum slow-phase velocity of the caloric test and LSCC VOR gain of the vHIT (correlation coefficient 0.792, *p* = 0.004). The CBI-absent group showed significantly lower SPV and LSCC VOR gains than the CBI-present group (*p* = 0.001 and 0.004, respectively).

**Discussion:**

LSCC dysplasia impairs VOR function, especially in the absence of CBI.

## 1 Introduction

Lateral semicircular canal (LSCC) dysplasia is among the most common inner ear malformations (1). In the sixth gestational week, semicircular canals (SCCs) develop as folded evaginations of the membrane from the vestibular appendage. Failure of this procedure results in the complete absence of SCCs. The central portion of this half-disc-shaped evagination is then resorbed and replaced by the mesenchyme, thus forming a semicircular duct. Incomplete absorption of this central membrane may result in a single fluid-filled cavity confluent with the vestibule. LSCC is the last of the three SCCs to form during the fetal period, making it particularly vulnerable to isolated abnormalities (2). Hypoplasia, such as abnormally large osseous LSCC, is the most common isolated anomaly of LSCC (3). While many studies have been conducted on the hearing levels of patients with LSCC dysplasia, the association of LSCC dysplasia with vestibular function loss still requires further understanding, with only a few studies published on the topic (4, 5). Dizziness is uncommon in patients with LSCC (2, 6). Valvassori et al. (6) emphasized that an abnormality in the bony canal of the inner ear does not necessarily indicate an abnormality of vestibular function. However, regardless of dizziness, most patients with unilateral LSCC dysplasia show abnormal values of canal paresis (CP) on the affected side in the caloric test (7). One previously suggested mechanism is that LSCC dysplasia may facilitate local endolymphatic circulation within the membranous labyrinth, weakening the hydrostatic pressure difference across the cupula and resulting in CP (8). In cases of dysplasia without a central bony island (CBI), which is the central portion of LSCC that should have been absorbed and replaced by bone, it is considered a more extensive form of dysplasia compared to cases of short and broad LSCC with normal or small CBI (9). We hypothesized that LSCC dysplasia without CBI would manifest as more severe CP in caloric tests than in those with CBI. This study aimed to compare the vestibular function in patients with LSCC dysplasia based on the presence of CBI. In addition, head impulse test results, which showed normal findings in LSCC dysplasia in previous studies, were analyzed (8, 10).

## 2 Materials and methods

### 2.1 Study population

We retrospectively evaluated the data of patients who presented with LSCC dysplasia following either temporal bone computed tomography (CT) or temporal magnetic resonance imaging (MRI) performed at our institution between May 2004 and October 2023. The patients were identified through a database query search of radiology reports. Imaging studies were primarily confirmed by a radiologist specializing in head and neck sections, and ENT specialists made the final diagnosis. Diagnosis of LSCC dysplasia was made when the area of the CBI was <7 mm^2^ (11). Patients who underwent vestibular function tests (caloric and/or video-head impulse tests) were included. Patients assessed prior to the introduction of the vHIT device to our hospital in 2015 only had the caloric test results, while patients assessed after 2015 had both the caloric test and vHIT results. The vestibular evoked myogenic potential (VEMP) test results were also documented when they were available. Patients with central or peripheral diseases that could affect the results of vestibular function tests, such as a history of vestibular neuritis, Meniere's disease, or vestibular schwannoma, were excluded from the study. This study was reviewed and approved by the Institutional Review Board of Gangnam Severance Hospital, Yonsei University Health System (IRB No. 3-2023-0351). The same institution waived the requirement for written consent due to the retrospective design of this study.

### 2.2 Vestibular function test

The results of the bithermal caloric test and video-head impulse test (vHIT) were collected. Each ear was irrigated with a constant water flow at 30 and 44°C for 30 s for the caloric test while recording eye movements using an infrared video-based system. The maximum slow-phase velocity (SPV) of nystagmus was calculated following each irrigation, and the Jongkees formula was used to determine CP (12). A CP of 25% or greater was considered abnormal. Bilateral canal paresis was defined when the sum of the maximal bithermal peak SPV on each side was <6°/s (13).

A vHIT was performed using a portable high-frame-rate video-oculography device (ICS Impulse, Otometrics, Denmark) consisting of lightweight infrared goggles with built-in rate and acceleration sensors. The patients were instructed to visually fixate on a laser dot on a screen at a distance of 90 cm, and ~20 horizontal head impulses were manually applied to each side with unpredictable timing and direction. The peak head velocity of the impulses was maintained between 150°/s and 200°/s. The mean vestibulo-ocular reflex (VOR) gains, which were automatically calculated, were used as parameters.

The cervical and ocular vestibular-evoked myogenic potential (cVEMP and oVEMP) responses were recorded in the ipsilateral sternocleidomastoid muscle (cVEMP) or the contralateral inferior oblique ocular muscle (oVEMP) by 95 dB HL, 500 Hz tone burst stimulation (ABaer, Natus Medical, Inc., CA, U.S.A). Those tests were performed by the protocols used in the previous report (14).

### 2.3 Statistical analysis

Spearman's correlation analysis was used to investigate the relationship between the SPV of the caloric test and the VOR gain of the vHIT. The groups' SPVs and LSCC VOR gains were compared using the Mann–Whitney *U*-test. Fisher's exact test was used to evaluate proportional significance. Results are expressed as the median [interquartile range (IQR)]. A *p*-value of < 0.05 was considered statistically significant. Statistical analyses were conducted using the SPSS software ver. 26 (IBM, Armonk, NY, USA).

## 3 Results

### 3.1 Patient characteristics

**Figure 1:**
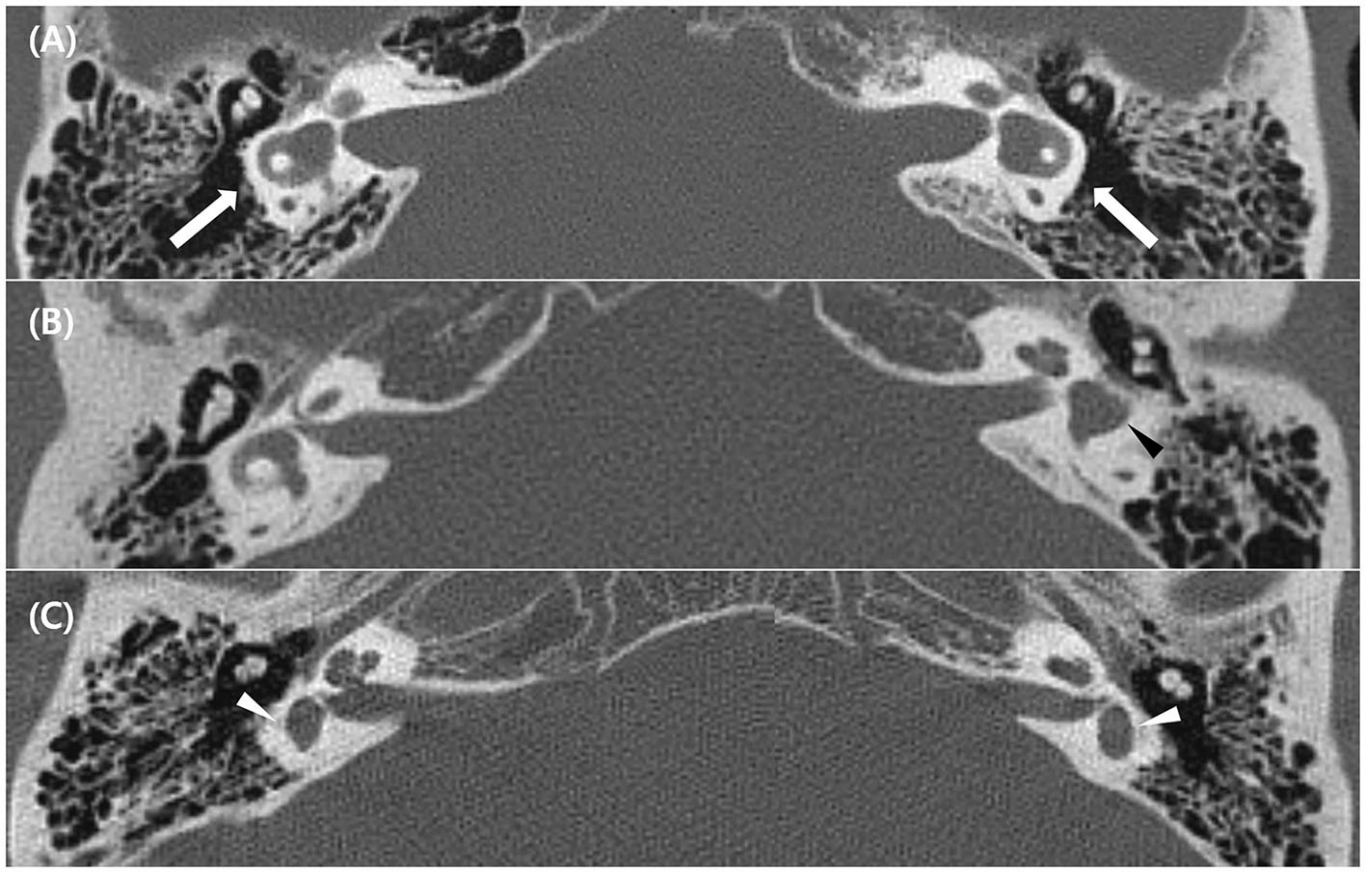
Temporal bone computed tomography (TBCT) image of the lateral semicircular canal (LSCC) dysplasia. **(A)** Bilateral LSCC hypoplasia with dilated SCC and enlargement of the vestibule (white arrows) (Patient 2 in Table 1). **(B)** Unilateral LSCC hypoplasia without a central bony island (black arrowhead) (Patient 8 in Table 1). **(C)** Bilateral LSCC aplasia with enlarged vestibule only (white arrowheads) (Patient 4 in Table 1).

Twenty patients with LSCC dysplasia (aplastic or hypoplastic) were identified. Among them, one was excluded due to the absence of vestibular function tests, and two had vestibular schwannoma on the affected side. Finally, 17 patients were included in this study (Table 1). Among them, eight were males and nine were females; their mean age was 51.9 ± 18.3 (age range, 16–80). LSCC dysplasia was observed unilaterally in 11 patients and bilaterally in six patients. Four of six patients with bilateral LSCC dysplasia showed combined inner ear anomalies; in contrast, only one of 11 patients with unilateral LSCC dysplasia had combined inner ear abnormalities. Among the 23 affected ears, two demonstrated aplasia, and 21 showed hypoplasia of the LSCC. One patient (no. 3) showed diffuse narrowing of the LSCC with combined narrowing of the superior and posterior SCCs. Other patients with LSCC hypoplasia demonstrated dilatation of the LSCC with small or absent bony islands. Nine of 23 ears had CBIs, and 14 ears had no CBI (Figure 1).

**Table 1:** Clinical characteristics of patients with lateral semicircular canal (LSCC) dysplasia.

**Patient no**.	**Sex**	**Age**	**Site of dysplasia**	**Lateral semicircular canal**	**Central bony island**	**Comorbid ear disease**
1	M	46	Both	Hypoplasia	No	No
2	F	59	Both	Hypoplasia	Yes	Incomplete partition II (both)
3	M	29	Both	Hypoplasia	Yes	Thin semicircular canals (both) Hypoplastic cochlea (both)
4	M	34	Both	Aplasia	No	ASCC/PSCC aplasia (both)
5	F	16	Both	Hypoplasia	Yes	Enlarged vestibular aqueduct (both)
6	F	67	Both	Hypoplasia	Yes (right) No (left)	Chronic otitis media on the right side
7	M	80	Left	Hypoplasia	No	No
8	F	59	Left	Hypoplasia	No	No
9	F	52	Left	Hypoplasia	No	No
10	F	47	Left	Hypoplasia	No	No
11	F	21	Left	Hypoplasia	No	Incomplete partition II (both) Enlarged utricle (right)
12	F	68	Right	Hypoplasia	Yes	No
13	M	45	Right	Hypoplasia	No	No
14	F	70	Right	Hypoplasia	No	No
15	M	70	Right	Hypoplasia	No	No
16	M	57	Right	Hypoplasia	No	No
17	M	62	Right	Hypoplasia	Yes	No

ASCC, anterior semicircular canal; PSCC, posterior semicircular canal.

### 3.2 Vestibular findings

**Figure 2:**
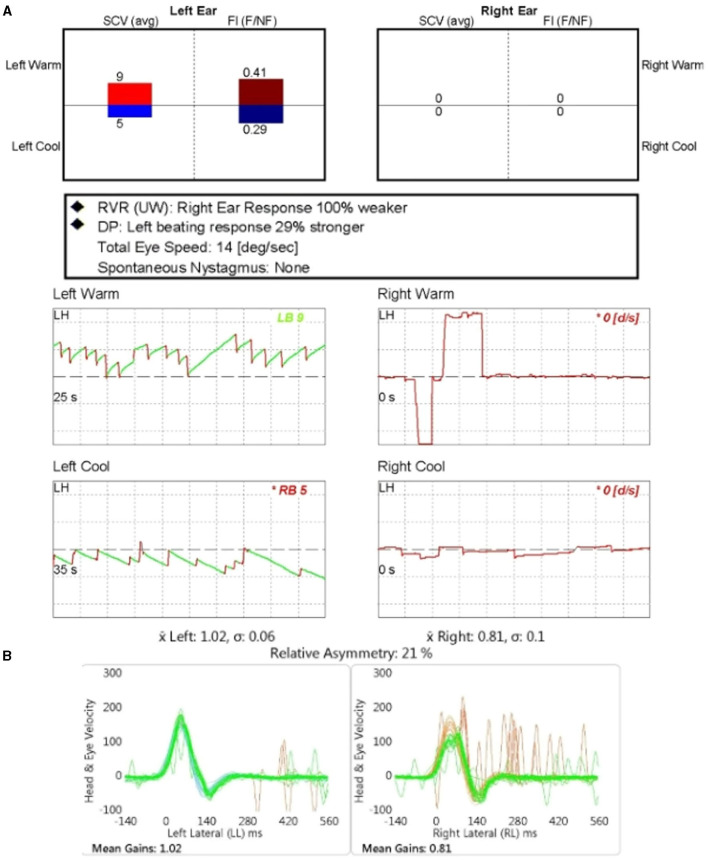
The results of the caloric test and video-head impulse test (vHIT) for patient number 7. **(A)** The caloric test revealed the unilateral canal paresis of 100% on the affected ear (right). **(B)** The vHIT gain of lateral semicircular canal in the affected ear (right) was reduced compared to the unaffected side.

Descriptions of dizziness and the results of the vestibular function tests are summarized in Table 2. Three of 17 patients experienced dizziness. Interestingly, all the patients with dizziness were in the CBI-absent group. One patient described the feeling of a spinning sensation; in contrast, the other two complained of chronic gait disturbances without a history of acute vertigo. The symptoms occurred during adulthood in all patients. Sixteen patients underwent caloric tests, and 12 patients underwent vHIT. Eleven of the 17 patients underwent caloric tests and vHIT. An example of results of a patient tested with caloric and vHIT is shown in Figure 2. Abnormal caloric test results were found in 11 of 16 patients (69%). All the patients with dizziness had abnormal caloric levels. Unilateral caloric CP was observed in nine of 16 patients, and bilateral CPwas observed in two. In the unilateral LSCC dysplasia group (*n* = 11), five patients (45%) showed correspondence of the side of CP with that of LSCC dysplasia; two (18%) did not show correspondence, three (27%) showed normal CP values, and one lacked caloric test results. In the bilateral LSCC dysplasia group (*n* = 6), two patients (33%) showed bilateral CP, two (33%) showed unilateral CP, and two (33%) showed normal CP values. Among the two patients with bilateral CP, one had aplasia in all SCCs (Patient 4), and the other had diffuse narrowing in all SCCs (Patient 3). Except for the case involving the narrowing of all SCCs, LSCC VOR gains exceeding 0.8, a threshold typically considered normal, were observed in 11 out of 12 patients (15). In addition, the VOR gains of anterior semicircular canal (ASCC) and posterior semicircular canal (PSCC) all exceeded the normal cut-off value of 0.7 for vertical canals (16, 17). Among the total of 23 ears, 14 ears had available vHIT results, and the mean (standard deviation) of VOR gains were as follows: LSCC 0.91 ± 0.04, ASCC 0.90 ± 0.05, PSCC 1.02 ± 0.07, and the mean of three SCCs 0.94 ± 0.04.

**Table 2:** Vestibular symptoms and functions in patients with LSCC dysplasia.

**Patient no**.	**Site of dysplasia**	**Central bony island**	**Dizziness symptom**	**Onset age of dizziness (yr)**	**Dizziness feature**	**SPV (°/s) (Rt/Lt)**	**Side of CP**	**CP (%)**	**Horizontal vHIT gain (Rt/Lt)**
2	Both (hypoplasia)	Yes	No			42/39	Lt	3	
3	Both (narrowing)	Yes	No			0/0	B	100	0.67/0.71
5	Both (hypoplasia)	Yes	No			19/14	Lt	15	1.09/1.01
12	Rt (hypoplasia)	Yes	No			115/57	Lt	33	1.14/1.09
17	Rt (hypoplasia)	Yes	No			27.9/38.8	Rt	16.4	1.01/0.9
6	Both (hypoplasia)	Yes (Rt) No (Lt)	Yes	65	Gait imbalance	33.2/12.4	Lt	45	
1	Both (hypoplasia)	No	Yes	46	Spinning sense	5/9	Lt	31	
4	Both (aplasia)	No	No			0/0	B	100	
7	Lt (hypoplasia)	No	No			9/0	Lt	100	0.83/0.89
8	Lt (hypoplasia)	No	No			12/14	Rt	9	1/0.85
9	Lt (hypoplasia)	No	No			(–)	(–)	(–)	1.11/0.85
10	Lt (hypoplasia)	No	No			6/17	Rt	48	1.02/0.94
11	Lt (hypoplasia)	No	No			12/0	Lt	100	1.03/0.86
13	Rt (hypoplasia)	No	No			8/31	Rt	59	
14	Rt (hypoplasia)	No	No			31/36	Rt	7	0.94/0.94
15	Rt (hypoplasia)	No	Yes	68	Gait imbalance	10/34	Rt	55	0.92/0.92
16	Rt (hypoplasia)	No	No			0/14	Rt	100	0.81/1.02

CBI, central bony island; SPV, slow-peak velocity; CP, canal paresis; LSCC, lateral semicircular canal; vHIT, video-head impulse test; VEMP, vestibular evoked myogenic potential.

In total of 23 ears, cVEMP results were available for 17 ears, and among them, 10 ears exhibited an absent response in VEMP. The results of oVEMP were available for four ears, with all of them demonstrating absent response.

**Figure 3:**
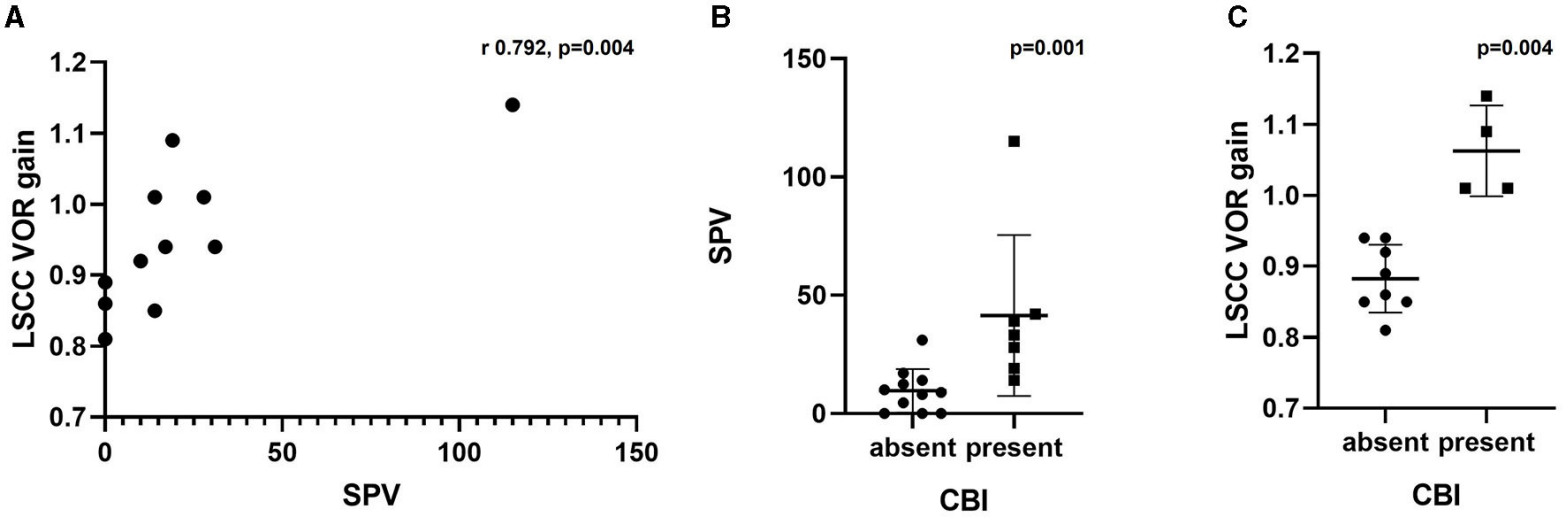
**(A)** Correlation between SPV in caloric test and LSCC VOR gain in vHIT. **(B)** Comparison of SPV in caloric test based on the presence of CBI. **(C)** Comparison of LSCC VOR gain in vHIT based on the presence of CBI. SPV, slow-phase velocity; LSCC, lateral semicircular canal; VOR, vestibulo-ocular reflex; vHIT, video-head impulse test; CBI, central bony island.

The correlation between the SPV of the caloric test and LSCC VOR gain of the vHIT was analyzed. Eleven patients with the results of both tests were included in the analysis. A significant correlation was identified between these variables, with a correlation coefficient of 0.792 (*p* = 0.004) (Figure 3A). However, the mean vHIT gain of the three SCCs did not correlate with the caloric test's SPV (*p* = 0.111).

The ears were divided into two groups according to the presence or absence of CBI to compare the vestibular test results (Table 3). One ear was excluded because of the lack of caloric test results. Patients with LSCC dysplasia and narrowed LSCC (Patients 3 and 4) who showed bilateral CPwere excluded from this subset analysis. Therefore, 18 ears were divided into two groups, with 11 ears in the CBI-absent group and seven in the CBI-present group. We compared the SPV of the caloric test and the LSCC VOR gain of the vHIT in the affected ear between the two groups. The CBI-absent group showed significantly lower SPV compared to the CBI-present group (*p* = 0.001), with a median value of 33.2 (IQR 19.0–42.0) and 8.9 (IQR 0–14.0), respectively (Figure 3B). The VOR gain in the LSCC was also significantly different between the two groups (*p* = 0.004); however, all values were within the normal range. The median gain of the CBI-absent group was 0.88 (IQR 0.85–0.94), and that of the CBI-present group was 1.05 (IQR 1.01–1.13) (Figure 3C). However, The VOR gain of ASCC and PSCC showed no difference between the two groups (*p* = 0.0.667 and 0.648, respectively).

**Table 3:** Comparison of groups according to the presence of central bony island.

	**CBI-present ears**	**CBI-absent ears**	***p*-value**
*N*	7	11	
Sex (M:F)	1:6	6:5	0.151^b^
Age	59.0 (IQR 16.0–67.0)	57.0 (IQR 46.0–70.0)	0.791^a^
Side (Rt:Lt)	5:2	5:6	0.367^b^
Caloric SPV	33.2 (IQR 19.0–42.0)	8.9 (IQR 0–14.0)	0.001^a*^
*N*	4	8	
vHIT LSCC gain	1.05 (IQR 1.01–1.13)	0.88 (IQR 0.85–0.94)	0.004^a*^

a Mann–Whitney *U*-test.

b Fisher exact test.

* *p* < 0.05.

CBI, Central bony island; SPV, slow-phase velocity; LSCC, lateral semicircular canal; IQR, interquartile range.

## 4 Discussion

Although LSCC dysplasia is the most frequently observed inner ear malformation (1), its relationship with hearing loss or vertigo remains unclear (5, 18). Previous studies have reported vestibular symptoms in 19.0–26.7% of patients with LSCC dysplasia (2, 7). Since many cases of LSCC dysplasia are asymptomatic, detailed vestibular functions in these cases remain unevaluated. Of the 17 cases reported in this study, only three patients (17.6%) experienced dizziness. Dizziness in these three cases was likely to be unrelated to pre-existing anomalies because the symptoms occurred after middle age. Most patients were asymptomatic despite anomalies, suggesting they had the proper VOR function required for daily activities. However, abnormal caloric test results were detected in 11 of the 16 patients (69%); in contrast, most patients (92%) had normal LSCC VOR gain on vHIT.

The mechanism of caloric response is a temperature-generated density change in the LSCC endolymph that leads to convection flow within the endolymph, which then deflects the cupula, appearing as nystagmus (19). Dissociation between caloric and head impulse test results in patients with Meniere's disease has also been noted (20–22). The caloric test revealed impairment of VOR in the LSCC plane; in contrast, the vHIT of the LSCC showed a normal or near-normal gain. McGarvie et al. demonstrated that abnormal CP in the caloric test may result from theoretically dissipated hydrostatic pressure in the dilated membranous labyrinth in Meniere's disease (20, 23). They proposed that, in patients with Meniere's disease, hydropic expansion of the horizontal SCC membranous labyrinth allows for local convective flow and mixing of the lower- and higher-density endolymph induced by a thermal gradient across the temporal bone. Without a hydrostatic drive force across the cupula, deflection of the cupula would not occur during the caloric test, resulting in an impaired response. However, the radius of curvature of the entire canal remains unchanged. Furthermore, hydrops reduce the hydrodynamic flow resistance in the duct, thus allowing a larger cupula deflection during angular rotation, theoretically resulting in a slight increase in VOR gain (24, 25).

Several vestibular function tests in patients with LSCC dysplasia have been previously reported (8, 10). In these studies, the caloric test revealed significant CP on the dysplasia side; in contrast, vHIT showed a normal gain. They explained this dissociation by applying a theoretical dissipation model, which was applied to Meniere's disease. They proposed that a common cavity formed by the vestibule and SCC enables active local endolymphatic circulation within the membranous labyrinth, leading to a loss of the hydrostatic pressure difference across the cupula. For a normal VOR gain, it has been claimed that this anomaly does not affect the rotational test. This interpretation assumes that the actual VOR function of patients with LSCC dysplasia does not decrease but that this anatomic anomaly decreases the thermal gradient in the caloric response. However, in this study, we recruited more patients and analyzed the caloric responses of the groups with and without CBI. The CBI-absent group showed significantly lower SPV and VOR gains than the CBI-present group. Furthermore, we found a significant correlation between the SPV of the caloric test and LSCC VOR gain in the vHIT. Patients with a more severe CP in the caloric test were likely to have a lower VOR gain in the vHIT. However, although the VOR gain of patients in the CBI-absent group was within the normal range, the VOR gain of the CBI-present group was slightly higher than that of the normal group.

Previous studies have attempted to explain this phenomenon by using endolymphatic hydrops. Endolymphatic hydrops in patients with LSCC dysplasia have been evaluated in several studies. The size of the endolymph was larger in the dysplasia group than in the normal group, and a strong negative correlation was found between the CBI area and the size of the endolymph (26). Therefore, endolymphatic hydrops is supposed to exist in LSCC dysplasia, and hydrops may contribute to increased local convection flow during caloric tests and reduced hydrodynamic flow resistance during vHIT, resulting in decreased SPV and slightly increased VOR gain, respectively. However, this is not the only reason for the abnormal caloric test results, as we found a strong correlation between SPV and VOR gain. This implies that contrary to previous studies, LSCC dysplasia debilitates VOR function, especially in the absence of CBI. The fact that all the patients who experienced dizziness belonged to the CBI-absent group also supports this conclusion. The normal VOR gain in the CBI-absent group can be explained by the fact that the deterioration in VOR function was neutralized by a slight increase due to the endolymphatic hydrops effect. As cupula displacement during rotation is affected by the average inverse cross-sectional area of the canal lumen, the absence of CBI would impede cupula displacement owing to the expanded cross-sectional area (27). It is also assumed that inefficient endolymph flow occurs as the streamline formed around the CBI disappears.

There are some limitations in this study. Because of the retrospective nature of this study, certain tests could not be collected for every patient. Moreover, the small sample size due to the low incidence of this congenital anomaly may affect the results obtained in our study. However, to our knowledge, this study has the largest number of subjects among the previous reports regarding LSCC dysplasia and vestibular function.

In conclusion, a more severe form of LSCC dysplasia, including the absence of CBI, was associated with a worse VOR in the caloric test. The vHIT results were mostly within the normal range; however, the gain in vHIT was also affected by the severity of LSCC dysplasia and correlated with caloric SPV. The VOR in LSCC dysplasia was not as normal as previously reported.

## Data availability statement

The raw data supporting the conclusions of this article will be made available by the authors, without undue reservation.

## Ethics statement

The studies involving humans were approved by Yonsei University Gangnam Severance Hospital, Institutional Review Board. The studies were conducted in accordance with the local legislation and institutional requirements. The Ethics Committee/Institutional Review Board waived the requirement of written informed consent for participation from the participants or the participants' legal guardians/next of kin because this study was conducted by retrospective review of medical records.

## Author contributions

JY: Data curation, Formal analysis, Investigation, Project administration, Software, Visualization, Writing – original draft. SK: Resources, Investigation, Writing – review & editing. SB: Conceptualization, Funding acquisition, Methodology, Resources, Supervision, Validation, Writing – review & editing.
